# Short-Term Effects of Climatic Variables on Hand, Foot, and Mouth Disease in Mainland China, 2008–2013: A Multilevel Spatial Poisson Regression Model Accounting for Overdispersion

**DOI:** 10.1371/journal.pone.0147054

**Published:** 2016-01-25

**Authors:** Jiaqiang Liao, Shicheng Yu, Fang Yang, Min Yang, Yuehua Hu, Juying Zhang

**Affiliations:** 1 West China School of Public Health, Sichuan University, Chengdu, Sichuan Province, China; 2 Office of Epidemiology, Chinese Center for Disease Prevention and Control, Beijing, China; Columbia University, UNITED STATES

## Abstract

**Background:**

Hand, Foot, and Mouth Disease (HFMD) is a worldwide infectious disease. In China, many provinces have reported HFMD cases, especially the south and southwest provinces. Many studies have found a strong association between the incidence of HFMD and climatic factors such as temperature, rainfall, and relative humidity. However, few studies have analyzed cluster effects between various geographical units.

**Methods:**

The nonlinear relationships and lag effects between weekly HFMD cases and climatic variables were estimated for the period of 2008–2013 using a polynomial distributed lag model. The extra-Poisson multilevel spatial polynomial model was used to model the exact relationship between weekly HFMD incidence and climatic variables after considering cluster effects, provincial correlated structure of HFMD incidence and overdispersion. The smoothing spline methods were used to detect threshold effects between climatic factors and HFMD incidence.

**Results:**

The HFMD incidence spatial heterogeneity distributed among provinces, and the scale measurement of overdispersion was 548.077. After controlling for long-term trends, spatial heterogeneity and overdispersion, temperature was highly associated with HFMD incidence. Weekly average temperature and weekly temperature difference approximate inverse “V” shape and “V” shape relationships associated with HFMD incidence. The lag effects for weekly average temperature and weekly temperature difference were 3 weeks and 2 weeks. High spatial correlated HFMD incidence were detected in northern, central and southern province. Temperature can be used to explain most of variation of HFMD incidence in southern and northeastern provinces. After adjustment for temperature, eastern and Northern provinces still had high variation HFMD incidence.

**Conclusion:**

We found a relatively strong association between weekly HFMD incidence and weekly average temperature. The association between the HFMD incidence and climatic variables spatial heterogeneity distributed across provinces. Future research should explore the risk factors that cause spatial correlated structure or high variation of HFMD incidence which can be explained by temperature. When analyzing association between HFMD incidence and climatic variables, spatial heterogeneity among provinces should be evaluated. Moreover, the extra-Poisson multilevel model was capable of modeling the association between overdispersion of HFMD incidence and climatic variables.

## Introduction

Hand, Foot, and Mouth Disease (HFMD) is an infectious disease that is mainly caused by a spectrum of pathogens in the enterovirus family. HFMD typically affects infants and children under 5 years old. However, it can also affect adults. Transmission occurs from person to person through direct contact with saliva, feces, vesicular fluid, or respiratory droplets from an infected person or indirectly via contaminated articles. Typically, the incubation period of HFMD is one week [1].

HFMD was initially reported in New Zealand in 1957 and then became frequently reported worldwide. Large outbreaks of HFMD caused by enterovirus 71 have been reported in East and Southeast Asia since 1970. Singapore first reported an outbreak of HFMD in 1970 [2]. Japan reported outbreaks in 1978 [3], and more outbreaks have been reported in Malaysia and South Korea since 1990 [4, 5]. China first reported an outbreak of HFMD in 1981 in Shanghai, and recent outbreak areas include Linyi city of Shandong province [6], Fuyang city of Anhui province [7] and Guangdong province [8]. Qi Zhu et al. (2011) [9] found that more than 1065000 cases of HFMD were reported in Mainland China from May 2008 to December 2009 and that the highest HFMD incidence were detected in Beijing city, Shanghai city, Zhejiang province and Hainan province. A recent study [10] also found that the morbidity of HFMD in China increased from 37.6/100 000 in 2008 to 139.6/100 000 in 2014 and most of the survivors were found in southern and eastern of China. County-level spatial temporal cluster analysis has been applied to Sichuan province, Liaocheng city of Shandong province, Guangdong province and Guangxi province [11–14] and has detected most likely spatial-temporal clusters. However, few studies have explored the risk factors which caused those spatial-temporal heterogeneity.

Numerous studies have found a strong association between climatic variables and HFMD incidence [15–17]. The associated climatic variables include humidity, temperature, rainfall, and wind speed. Mitsuyoshi (2003) found that the climatic variables and calendar months explained 64% of the variation of HFMD incidence in Tokyo [16]. Hii YL, Rocklov J, and Ng N (2011) found that weekly temperature and rainfall were significantly associated with HFMD incidence and lag effects were detected within 2 weeks in Singapore [15]. Studies in China also found that temperature, humidity and sunshine were risk factors for HFMD [18, 19]. However, those studies mainly focused on one province or one city and lack of provincial level analysis for China. Those studies used logistic regression, time series models, generalized additive models or distributed lag models, none of which accounted for both the spatial and temporal heterogeneity between HFMD incidence and climatic variables.

The aim of this study is to examine the short-term association between weekly HFMD incidence and weekly climatic variables and to explore provincial spatial heterogeneity between HFMD incidence and climatic variables.

In this study, we first checked the serial autocorrelation of weekly HFMD cases to determine whether lag effects should be included. Second, we explored the lag periods and nonlinear association between climatic variables and HFMD incidence. Finally, we analyzed the provincial level short-term association between climatic variables and HFMD incidence. Provincial spatial heterogeneity associated with climatic variables was evaluated by comparing the provincial relative risk(*RR*) of climatic variables.

## Data and Methods

### Data sources

The weekly number for the period 2008–2013 of HFMD cases in 31 provinces of Mainland China for the period of 2008–2013 was obtained from the China Information System for Disease Control and Prevention (CISDCP) supported by the Chinese Center for Disease Control and Prevention. The clinical diagnostic criteria for HFMD cases was based on a guidebook published by the Ministry of Health of the People’s Republic of China in 2008 [20]. The climatic data on daily average temperature, daily maximum temperature, daily minimum temperature, 24–24 hours of rainfall, daily hours of sunshine, daily relative humidity and daily average wind speed were obtained from the China Meteorological Data Sharing Service System (http://cdc.cma.gov.cn/home.do). Daily climatic variables were averaged to create weekly mean values. The weekly climatic data at the provincial level were then interpolated using Kriging methods [21]. The annual populations of provinces were obtained from the 2008 to 2013 China Statistical Yearbooks.

### Statistical analysis

The white noise test was used to evaluate the serial correlation of the weekly HFMD cases. Weekly time trend plots were used to compare HFMD cases and climatic variables between 2008 and 2013. Provincial HFMD incidence were calculated by the reported HFMD cases divided by the annual provincial population. The polynomial distributed lag model was used to analyze the lag effects between climatic variables and HFMD incidence. The statistically significant climatic variables tested by polynomial distributed lag model were further explored by smooth spline plot.

A multilevel polynomial extra-Poisson regression model was used to model cluster effects for provinces and years. The significant nonlinear terms and lag effect terms tested by the polynomial distributed lag model were included in the multilevel extra-Poisson model. Provincial annual population was included in the multilevel extra-Poisson regression model as an offset variable. We also included a conditional normal distribution random effects at the province level to account for spatial auto-correlative relationship between adjacent provinces. We fitted those multilevel statistical models using Mlwin 2.32(Sichuan University, China) and WinBUGS 14 by MCMC methods and results for the model with the smallest *DIC* values were reported.

### Polynomial distributed lag model

The basic polynomial distributed lag model was constructed as follows:

$$\begin{array}{cc} Y_{t} & =\alpha +\beta_{0}X_{t}+\beta_{1}X_{t-1}+\ldots +\beta_{i}X_{t-i}+e_{t}\\ \beta_{i} & =\alpha_{0}+\sum_{j=1}^{d}\alpha_{j}i^{j} \end{array}$$

*Y*_*t*_ denotes the weekly reported HFMD cases adjusted by annual population.*X*_*t*−*i*_ denotes lag *i* periods of climatic variables *X*_*t*_. *α* denotes the baseline effect when all covariates equal 0. *β*_*i*_ denotes lag coefficients and follows a polynomial in the lag periods *i*. The distribution of the lag effects is modeled by lag polynomials $\sum_{j=1}^{d}\alpha_{j}i^{j}$, *d*(≤*p*) denoting the degree of the polynomial. *e*_*t*_ denotes residual error. The weeks of lag effects were selected according to the related literatures and statistically significant lag effects terms tested by Polynomial distributed lag model. The minimal statistically significant weeks of lag periods were included in the multilevel extra-Poisson model.

To further explore the natural effects between HFMD incidence and climatic variables, the significant climatic variables tested by polynomial distributed lag model were plotted by HFMD incidence with smooth spline function.

### Multilevel extra-Poisson regression model

Multilevel extra-Poisson models were used to model the association between weekly climatic variables and HFMD incidence. Weekly average climatic variables (rainfall, temperature, wind speed, and hours of sunshine), the polynomial terms of climatic variables and the lag effects detected in a polynomial distributed lag model were included. To adjust for the long period trend, the year variable was coded into 5 dummy variables, and the year of 2008 was treated as a reference. A three level poisson model was constructed. Specific definitions for the hierarchy include:

1. level 3:provinces,denoted by *k*
2. level 2:years,denoted by *j*
3. level 1:weeks,denoted by *i*

Details of the model were introduced as follows:

*pcase*_*ijk*_ ∼ *poisson*(λ_*ijk*_)
$\begin{array}{cc} & \log\left(\lambda_{ijk}\right)=\ln\left(pop_{ijk}\right)+\beta_{0ijk}+\sum_{m=1}^{m}\beta_{mk}X_{mijk}+\sum_{n=1}^{n}\beta_{n}{X_{t-n}}_{ijk}+\sum_{p=1}^{p}\beta_{p}X_{pijk}^{p}\\ & +\sum_{year=1}^{5}\beta_{year}X_{year} \end{array}$
$\beta_{0jk}=\beta_{0}+v_{0k}+u_{0jk}+u_{province[i]}^{\left(2\right)}$
*β*_*mk*_ = *β*_*m*_ + *v*_*mk*_
$\left[\begin{array}{c} v_{0k}\\ v_{mk} \end{array}]∼N\left(0,\Omega_{v}\right)\;:\Omega_{v}=[\begin{array}{cc} \sigma_{v0}^{2} & \\ \sigma_{v0m} & \sigma_{vm}^{2} \end{array}\right]$
$\left[\mu_{0jk}]∼N\left(0,\Omega_{u}\right)\;:\Omega_{u}=[\sigma_{u0}^{2}\right]$
$u_{{}_{province[k]}}^{\left(2\right)}∼N\left({\bar{u}}_{{}_{province[k]}}^{\left(2\right)},\sigma_{u(2)}^{2}/w_{province[k]}\right)$
${\bar{u}}_{{}_{province[k]}}^{\left(2\right)}=\sum_{j\in neighbour(province[k])}c_{{}_{province[k],j}}^{\left(2\right)}u_{j}^{\left(2\right)}/w_{province[k]}$
*var*(*pcase*_*ijk*_|*λ*_*ijk*_) = *αλ*_*ijk*_

*pcase*_*ijk*_ denotes HFMD cases of week *i* in *j* year of province *k*.*λ*_*ijk*_ denotes the expected cases based on a Poisson distribution. *ln*(*pop*_*ijk*_) denotes an offset for weekly HFMD cases adjusted by log transformation of the annual population of provinces.*β*_0_ denotes intercept effects without adjustment for climatic variables. *β*_*m*_ denotes the linear fixed effects of climatic variables.*β*_*n*_ denotes the *t* − *k* periods of lag effects of climatic variable *X*_*t*_. *β*_*p*_ denotes the effects of polynomial of climatic variables.*β*_*year*_ denotes the effects of dummy variables of years 2009–2013.*v*_0*k*_ denotes the intercept random effect for province *k*, *μ*_*ojk*_ denotes the intercept random effect for province *k* in *j* year.*v*_*mk*_ denotes the random effects of *m* climatic variables for province *k*. $u_{province[k]}^{\left(2\right)}$ denotes the spatial random effects of province *k* based on a conditional normal distribution with mean equal to ${\bar{u}}_{{}_{province[k]}}^{\left(2\right)}$ and variance equal to $\sigma_{u(2)}^{2}/w_{province[k]}$. $u_{j+}^{\left(2\right)}$ denotes the spatial random effects of province *j*+. $c_{{}_{province[k],j+}}^{\left(2\right)}$ denotes the adjacent indicator, if province *k* and *j*+ are adjacent then $c_{{}_{province[k],j+}}^{\left(2\right)}=1$, otherwise $c_{{}_{province[k],j+}}^{\left(2\right)}=0$. *w*_*province*[*k*]_ denotes the adjacent numbers of provinces for province *k*.*α* denotes a scale parameter that measures dispersion, and *α* > 1 indicates overdispersion. Prior-distributions were assumed to Gamma distribution for hyper-parameters of random effects:$1/\sigma_{v0}^{2}∼Gamma\left(0.001,0.001\right)$, $1/\sigma_{vm}^{2}∼Gamma\left(0.001,0.001\right)$, $1/\sigma_{u0}^{2}∼Gamma\left(0.001,0.001\right)$, $1/\sigma_{u(2)}^{2}∼Gamma\left(0.001,0.001\right)$.

## Results

### Autocorrelation analysis of overall HFMD cases

**Fig 1:**
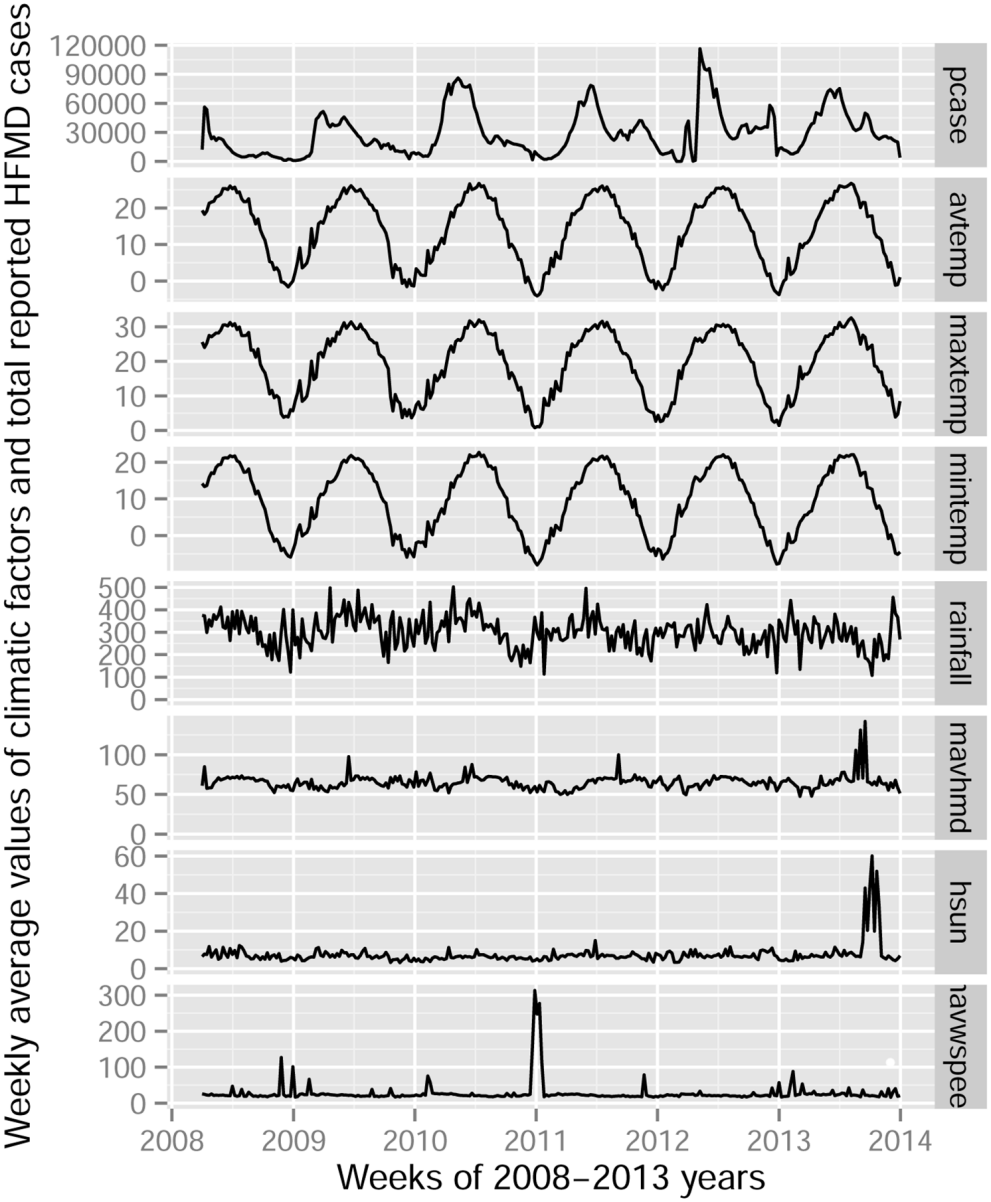
Time series plot of weekly HFMD cases and climatic variables of China, 2008–2013. Pcase (weekly reported cases of HFMD), avtemp (weekly average temperature (°C)), maxtemp (weekly maximum temperature (°C)), mintemp (weekly minimum temperature (°C)), rainfall (weekly average 20–20 hours rainfall (0.1 millimeters)), mavhmd (weekly average relative humidity (0.1%)), hsun (weekly average hours of sunshine (0.1hours)), mavwspeed (weekly average wind speed (meters per second)).

The result of the white noise test was significant (*p* < 0.0001), indicating that the series data of HFMD cases were non-stationary and that an autoregressive model should be used. Fig 1 shows that the peak weeks of HFMD cases clustered are in weeks 17–23, and the number of HFMD cases is relatively high in 2011 and 2012. Temperature, humidity, and hours of sunshine may be associated with HFMD cases because their time trends were close to time trend of HFMD cases. A high correlation (correlation coefficient = 0.968) was found between maximum weekly temperature and minimum weekly temperature; thus, we calculated a temperature difference variable (devtemp) that calculated as the weekly maximum temperature minus the weekly minimum temperature.

### Results of smooth spline plot and the polynomial distributed lag model

The polynomials were tested to 5 orders and the lag effects were tested within 20 weeks. The significant results were listed in Table 1. For weekly association between avtemp and HFMD incidence, we detected significant non-liner effects at the 2nd and 4th order polynomial terms and detected significant lag effects at 2 weeks. For weekly association between devtemp and HFMD incidence, we detected significant non-linear effects at the 2nd polynomial terms and detected significant lag effects at 2 weeks. For weekly association between mavhmd and HFMD incidence, we detected significant non-linear effects at the 2nd order polynomial terms and detected significant lag effects at 3 weeks. For weekly association between hsun and HFMD incidence, we detected significant non-linear effects at the 2nd order polynomial terms.

**Table 1:** The significant (*p* < 0.05) polynomial terms and lag effects periods tested by polynomial distributed lag model.

**Variable**	**Polynomial terms**	**Lag effects terms**
Mavhmd	mavhmd**1,mavhmd**2	mavhmd(1),mavhmd(2),mavhmd(3)
Avtemp	avtemp**1,avtemp**2,avtemp**3,avtemp**4	avtemp(1),avtemp(2),avtemp(3)
Devtemp	devtemp**1,devtemp**2	devtemp(1),devtemp(2)
Hsun	hsun**2	-

X**n stands for high order terms of climate factors. X(n) stands for detecting weeks of lag periods in climate factors.

**Fig 2:**
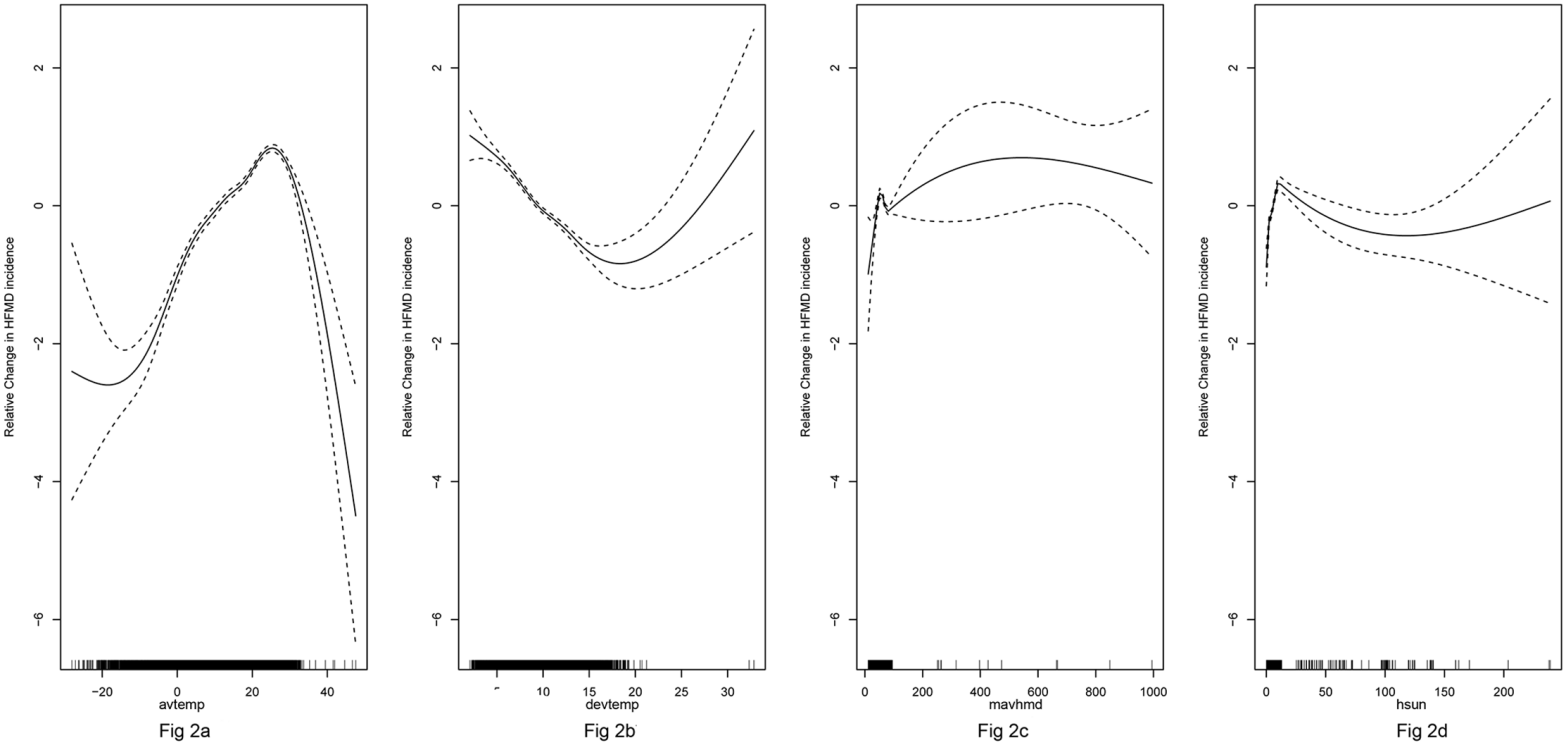
Relationship between climatic variables and HFMD incidence. Relative changes of HFMD incidence as smooth spline function of avtemp (2a), devtemp (2b), mavhmd (2c) and hsun (2d). (Solid line: fitted values; dash line: 95% confidence interval of fitted value; Vertical bar located on x-axis: distribution of climatic variables).

The natural exploration of HFMD incidence and significant climatic variables are listed in Fig 2. Fig 2a depicts an inverse “V” shaped relationship between HFMD incidence and avtemp, indicating every unit increase of avtemp below 27°C will increase HFMD incidence and every unit increase of avtemp beyond 27°C will reduce HFMD incidence. Fig 2b depicts a “v” shaped relationship between HFMD incidence and devtemp, indicating HFMD incidence decrease with increasing of devtemp below 17°C; whereas every unit increases of devtemp beyond 17°C increase HFMD incidence. Fig 2c depicts HFMD incidence increase with increase of mavhmd below 5% or range from 10% to 50%; whereas every unit increase of mavhmd from 5% to 10% or above 50% reduces HFMD incidence. Fig 2d depicts HFMD incidence increase with hsun below 2 hours or above 10 hours; whereas every unit increase of hsun from 2 hours to 10 hours reduces HFMD incidence.

### Results of the multilevel extra-Poisson regression model

Because the 3nd-order and 4nd-order polynomials of weekly average temperature are very weakly associated with HFMD incidence compared with the 2nd-order polynomial, we only kept the 2nd-order polynomial of weekly average temperature in multilevel extra-Poisson model. Summary of variables selection process were listed at Table 2.

**Table 2:** Summary results of model selection process.

**Variable**	**Variables**^a^	***DIC***
model1	avtemp	132594
model2	devtemp	136845
model3	rainfall	–^b^
model4	mavhmd	136718
model5	mavwspeed	136831
model6	hsun	136831
model7	avtemp+devtemp	130457
model8	avtemp+devtemp+mavhmd	130465
model9	avtemp+devtemp+mavwspeed+hsun	–^b^
model10(spatial structure)	avtemp+devtemp	130376
model11(spatial+random effcts of avtemp)	avtemp+devtemp	129858

^a^this climatic variable related polynomial terms and lag effects terms also included in the model.

^b^model was not converged and can’t be estimated.

Model 11 was selected as our final reported model based on smallest *DIC* value(Table 2) and results of model 11 were listed in Table 3. The results of the multilevel extra-Poisson regression model showed that the scale parameter was 548.077 indicating a big difference existed between mean of HFMD incidence and variance of HFMD incidence. The assumption of a Poisson distribution for HFMD incidence violated this relationship motivating use of the extra-Poisson model to track gaps between mean and variance of HFMD incidence. The significant results of the random effects for provinces and years implied HFMD incidence clustered among provinces and years. After adjustment for overdispersion and cluster effects of provinces and years, variables which were statistical significantly associated with HFMD incidence included years, avtemp, temp2, lag1temp, lag2temp, lag3temp and lag2devtemp.

**Table 3:** Results of multilevel extra-Poisson regression model.

**Variable**	**Unadjusted *RR*(95%*CI*)**	**Adjusted *RR*^a^(95%*CI*)**
Year		
2008	1	1
2009	1.696(1.410,2.143)	2.096(1.710,2.559)
2010	2.680(2.246,3.252)	3.413(2.712,4.162)
2011	2.268(1.854,2.749)	2.823(2.320,3.483)
2012	2.831(2.260,3.353)	3.369(2.769,4.149)
2013	2.229(1.813,2.818)	2.756(2.221,3.456)
Avtemp	1.077(1.075,1.079)	1.033(1.017,1.048)
Temp2	1.002(1.001,1.002)	0.999(0.998,0.999)
Lag1temp	1.082(1.080,1.084)	1.018(1.006,1.027)
Lag2temp	1.087(1.085,1.089)	1.019(1.012,1.027)
Lag3temp	1.088(1.086,1.090)	1.024(1.017,1.030)
Devtemp	0.993(0.982,1.003)	1.013(0.981,1.034)
Detmp2	0.998(0.998,0.999)	0.999(0.998,1.000)
Lag1devtemp	1.000(0.991,1.008)	0.989(0.978,1.000)
Lag2devtemp	1.028(1.019,1.036)	1.013(1.003,1.024)
**Random effects**		
Level 3(province)	-	
Unstructure		10.080(3.247)
Avtemp	-	3887.000(1191.000)
cov(Unstructure,avtemp)		142.000(56.530)
Structure		37.710(48.720)
Level 2(year)		
Intercept	-	5.792(0.719)
Level 1	-	
Scale	-	548.077(8.116)

^a^adjusted variables included other variables which were remained in the multilevel model.

The dummy variable of year demonstrated that HFMD weekly incidence increased from 2009 to 2013 compared with 2008. Avtemp was strongly and significantly associated with HFMD incidence after adjustment for nonlinear association and lag effects. A 1°C increase in weekly avtemp was associated with an average 3.3% (95% CI:1.7%-4.8%) increase in relative risk of weekly HFMD incidence. The significant lag periods between avtemp and weekly HFMD incidence were 3 weeks. A 1°C increase in current avtemp contributed to an average 1.8% (95%CI:0.6%-2.7%) increase in relative risk of weekly HFMD incidence in lag 1 week. A 1°C increase in current avtemp contributed to an average 1.9% (95%CI:1.2%-2.7%) increase in the relative risk of weekly HFMD incidence in lag 2 weeks. A 1°C increase in current avtemp contributed to an average 2.4%(95%CI:1.7%-3.0%) increase in the relative risk of weekly HFMD incidence in lag 3 weeks.

Temperature difference was significantly associated with HFMD incidence in lag 2 weeks after adjustment for linear trend and nonlinear association. A 1°C increase in current devtemp contributed to a 1.3% (95%CI:0.3%-2.4%) increase in the relative risk of HFMD incidence in lag 2 weeks.

The significant polynomial term of temp2 in the model shows that temperature had a nonlinear relationship with HFMD incidence. The significant *RR* with value less than 1 after adjustment for linear trend and lag effects indicated that avtemp and weekly HFMD incidence approximated an inverse “V” shape association. This association depicts an increase trend of HFMD incidence when avtemp is below 27°C and depicts a decrease trend of HFMD incidence when avtemp is above 27°C.

### Provinces level heterogeneity exploration

**Fig 3:**
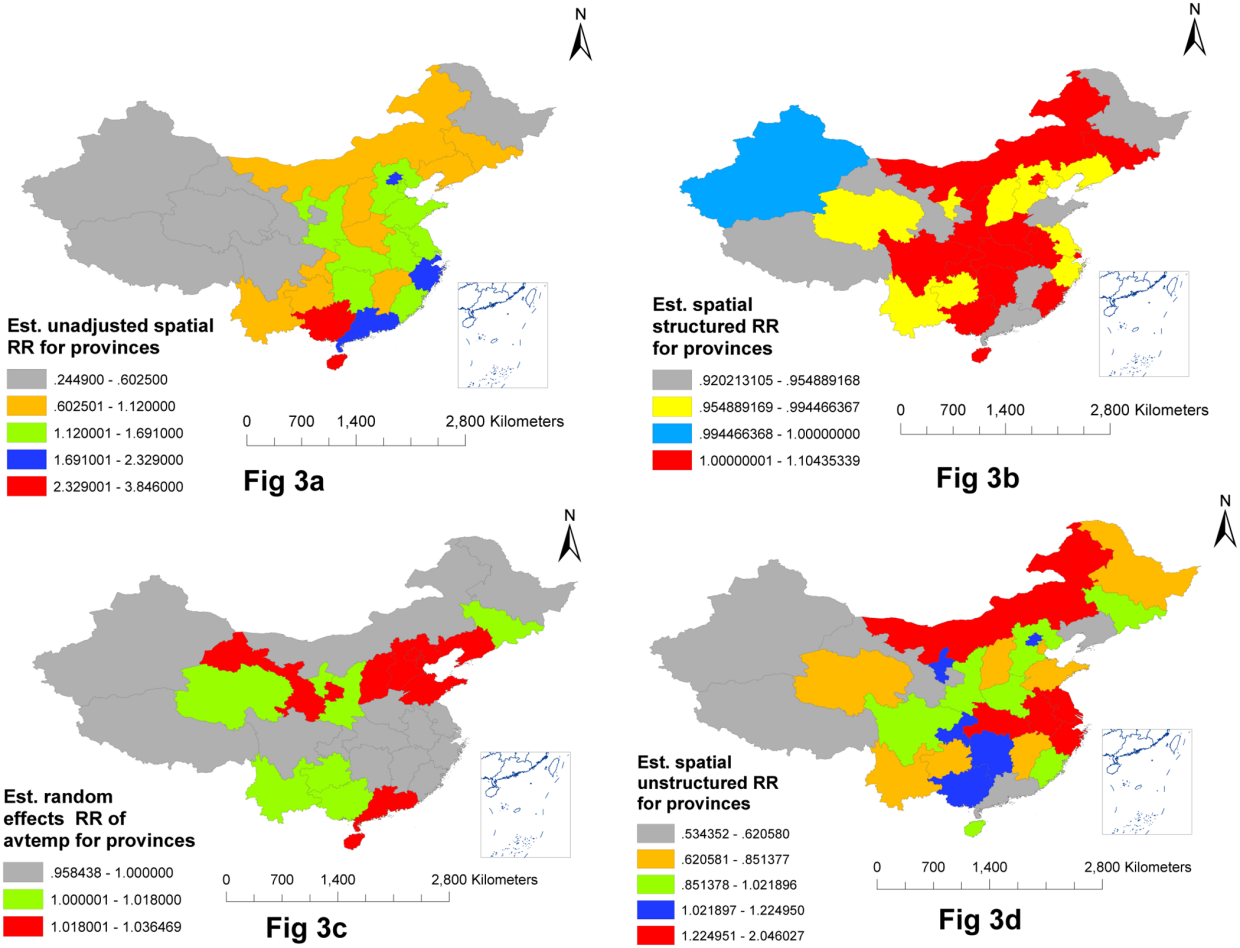
Estimated *RR* for province. Fig 3a. Estimated unadjusted spatial *RR* for provinces, Fig 3b. Estimated spatial structured *RR* for provinces, Fig 3c. Estimated random effects *RR* of avtemp for provinces, Fig 3d. Estimated spatial unstructured *RR* for provinces(Fig 3a *RR* generated from null multilevel model unadjustment for spatial correlated strucuture effects and climatic varialbes, Fig 3b–3d *RR* generated from final multilevel model adjustment for spatial correlated effects and climatic variables).

Fig 3a shows crude *RR* of HFMD incidence unadjusted for spatial correlation structure and climatic variables. The most highly unadjusted *RR* of HFMD incidence were detected in Guangxi province and Hainan province while the second most highly unadjusted *RR* of HFMD incidence were detected in Guangdong province, Zhejiang province, Beijing city and Tianjin city. Provinces located at northeastern, eastern, enteral and southeastern of China had estimated spatial structured *RR* above 1 which means HFMD incidence of those province were significantly spatial correlated compared with other provinces (Fig 3b). The association between avtemp and weekly HFMD incidence were different among provinces(Fig 3c). The weekly HFMD incidence was highly associated with avtemp in Hainan province, Guangdong province, Gansu province, Shanxi province, Hebei province, Shandong province, Beijing city, Tianjin city and Liaoning province. Zhejiang province, Jiangsu province, Anhui province, Hubei province, Inner Mongolia province, Guangxi province, Hunan province, Chongqing city, Ningxia province and Beijing city still had a high HFMD incidence after adjustment for climatic variables(Fig 3d).

## Discussion

Temperature is the main determinant of HFMD incidence in northeast and southeast China. The relatively strong association between avtemp and weekly HFMD incidence found in our study is consistent with other studies elsewhere in China. Yong Huang et al. [22] found a 1.86% increase in the weekly number of HFMD cases with a 1°C increase in temperature among 0–14 years old children in Guangzhou. Liu Li et al. [23] found a significant association between monthly reported HFMD cases and monthly average temperature in Shijiazhuang. Xu Yiling et al. [24] found that the monthly HFMD incidence (1/100000) decreased by 56.5% when the monthly average temperature increased by 1°C. Many studies have found lag effects within a few weeks or days between climatic variables and HFMD incidence [15, 22]. In our study, the significant lag periods for avtemp and devtemp were 3 weeks and 2 weeks. In addition, we further demonstrated a spatially heterogeneous association between avtemp and HFMD incidence among provinces and provinces located at northeastern, southeastern and southern of China also had a high association between avtemp and HFMD incidence.

The mechanism by which temperature affects HFMD incidence is complicated and requires further study. Numerous studies have found threshold effects between short-term changes in climate and incidence of HFMD [15, 22, 25]. Our findings showed that the risk function of weekly average temperature approximated an inverse V-shaped curve with HFMD incidence, and the threshold of weekly average temperature was 27°C. This association may be partially explained by the fact that temperatures between 2°C to 27°C offer a more sensitive environment for outbreaks of enterovirus [26], causing people to be more easily infected. Meanwhile high temperatures may be an obstacle for people to join social activities, making them less likely to be infected.

HFMD incidence were heterogeneously distributed in space and spread among provinces. Yu Shicheng et al. (2011) found that the patterns of HFMD incidence in Fujian, Hainan and Tibet were different from those in other provinces [27]. In our study, we found spatial heterogeneity clustered at the province level. We have identified mostly spatial correlated clusters in northern, northeastern, central and southeastern of China. Those correlations can be partially explained by variation of temperature in some southern and northeastern provinces such as Beijing city and Hainan province. Special cases were detected in Shanxi province and Hebei province. These provinces had a high association between avtemp and HFMD incidence and HFMD incidence was independent from other surrounding provinces.

We demonstrated high temperature can be used to explain high *RR* of HFMD incidence in southern and eastern province of China while temperature can’t be used to explain most of spatial spread of HFMD incidence. Factors such as socio-economic conditions or high HFMD susceptibility and their association with spatial correlated speared of HFMD should be further explored. Variation of HFMD incidence in Zhejiang province and Jiangsu province should also be explored as HFMD incidence in those provinces exhibited a low association with temperature, low spatial correlation yet still had high incidence.

Above all, provincial association between temperature and HFMD incidence can be categorized into three clusters. In the first cluster, high HFMD incidence, high association between HFMD incidence and temperature and variation of HFMD incidence can be mostly explained by temperature. This cluster included Hainan province, Guangdong province, Liaoning province, and Gansu province. In the second cluster, high HFMD incidence, low association with temperature, still had high variation after adjustment for temperature. This cluster included Shanghai city, Jiangsu province, Anhui province, Zhejiang province and Hubei province. The third cluster exhibited low HFMD incidence, Low association with temperature, low variation after adjustment for temperature. This cluster included Xinjiang province and Tibet province which could be partially explained by their lower amounts of rainfall or lower average temperatures [28]. Another reason for those findings may be that the surveillance systems in Xinjiang province and Tibet province were not well developed, and the newly infected cases of HFMD could not be reported in time [29].

Our findings are more efficient than previous studies which focused on pure spatial and temporal scan cluster analysis [11, 14, 17] because pure spatial temporal scan only detect highly clustered geographic units of HFMD incidence but can’t explore which factors are associated with those high clusters. In our research, we demonstrated 3 clusters of HFMD incidence. Most important, we decomposed spatial variation of HFMD incidence into spatial correlated component, random effects of temperature component and spatial unstructured component and found temperature was differently associated with those clusters. These findings can help with control of HFMD and measures should be taken to minimize socio activities when temperature is close to 27°C among temperature sensitive provinces while measures should be taken to minimize mobility of high susceptible risk of HFMD population in provinces with spatial correlated HFMD incidence.

In our study, we used a multilevel statistical model to address spatial heterogeneity. The multilevel statistical model treat the subgroup as a clustering effect and assumed the clustering effect as a random effect following a Gaussian distribution [30]. Additionally, we also modeled spatial correlated of HFMD incidence at the province level. The spatial correlated structured was assumed to follow a conditional normal distribution. After including spatial correlated structure component, *DIC* values of model fitting decreased significantly.

Overdispersion should be considered in our study. With category or count outcome data, in which variance may be much larger than the mean, overdispersion needs to be modeled [31]. Overdispersion will underestimate the standard errors, increasing the likelihood of obtaining a false positive result [32]. In our study, we found that the scale parameter measuring overdispersion was 548.077, indicating overdispersion. We used a multilevel extra-Poisson regression model to accommodate overdispersion. In contrast to the multilevel Poisson regression model, the multilevel extra-Poisson regression resulted in fewer significant associations with climatic variables, which were caused by overdispersion.

Some limitations of this study warrant mention. First, the infectious disease surveillance system may not capture all HFMD cases due to underreporting, especially in provinces located at western China [29]. Those lack of reporting HFMD cases may have led to an underestimation of HFMD incidence. Second, HFMD cases are characterized as fatal, severe and mild types, and these types might have different associations with climatic variables. Therefore, further research that differentiates specific types of HFMD cases are needed.

In conclusion, our study first clearly demonstrated that temperature exhibits a spatially heterogeneous association with provincial HFMD incidence in Mainland China. HFMD incidence in the southeastern and northeastern provinces of Mainland China is sensitive to temperature. Other factors should be further explored for northern, western and eastern provinces which exhibited a little association with temperature.
